# Dysphagia following cerebellar stroke: analyzing the contribution of the cerebellum to swallowing function

**DOI:** 10.3389/fneur.2023.1276243

**Published:** 2023-11-16

**Authors:** Masoume Hajipour, Davood Sobhani-Rad, Shahryar Zainaee, Mohammad Taghi Farzadfar, Saeedeh Hajebi Khaniki

**Affiliations:** ^1^Department of Speech Therapy, School of Paramedical and Rehabilitation Sciences, Mashhad University of Medical Sciences, Mashhad, Iran; ^2^Department of Communication Sciences and Disorders, College of Health and Human Services, Bowling Green State University, Bowling Green, OH, United States; ^3^Department of Neurology, Mashhad University of Medical Sciences, Mashhad, Iran; ^4^Department of Biostatistics, School of Health, Mashhad University of Medical Sciences, Mashhad, Iran

**Keywords:** cerebellum, cerebellar stroke, cerebellar infarct, dysphagia, swallowing disorders, deglutition disorders, swallowing, swallowing function

## Abstract

Swallowing is essential for human health, and the cerebellum is crucial for motor movement regulation. Cerebellar strokes may cause dysphagia, but their exact effects remain unexplored in swallowing function. Therefore, the aim of this study was to analyze the precise clinical characteristics of the oral and pharyngeal phases of swallowing after cerebellar stroke and to critically discuss the cerebellum’s contribution to swallowing. The study involved 34 participants with cerebellar strokes, gathered through convenience sampling. Neurologists diagnosed isolated strokes, and a speech and language pathologist examined swallowing ability using the Mann Assessment of Swallowing Ability. The study found that 52.9% of people experienced dysphagia after a cerebellar stroke. Dysphagia was significantly associated with a higher risk of aspiration. Age was also significantly correlated with dysphagia. No significant correlation was found between swallowing ability and sex. In conclusion, this study suggests isolated cerebellar stroke can adversely affect the motor and non-motor aspects of swallowing and cause severe dysphagia and aspiration risk. Thus, early diagnosis and timely management of dysphagia following a cerebellar stroke can help prevent serious consequences.

## 1 Introduction

Swallowing is a complex and basic mechanism allowing humans to eat and drink while preventing penetration or aspiration, which can result in pulmonary diseases (1). Swallowing, as Magendie described it first, is usually factored in as a process having three stages, including oral, pharyngeal, and esophageal (2, 3). This complex mechanism requires extreme coordination and arrangements among different body systems, such as the nervous system. Hence, different medical conditions of this system, such as stroke (4), multiple sclerosis (5), etc., can lead to swallowing disorders, also known as dysphagia.

The cerebellum, which makes up the majority of the hindbrain, is attached to the brainstem by three groups of cerebellar peduncles. The cerebellum has been acknowledged for its crucial involvement in the regulation of motor movement, postural maintenance, muscular tone control, balance, gait coordination, and volitional muscle activity. The cerebellar function appears to be only inhibitory (6). Indeed, the cerebellar cortex plays a crucial part in ensuring the accuracy, smoothness, and coordination of any muscular activity, even though it has no excitatory role and does not initiate movements (7, 8). The cerebellum also has many projections to the brainstem, such as nucleus tractus solitarius and nucleus ambiguous (9). The cerebellum is thought to affect different central pattern generators such as swallowing (10). Therefore, cerebellar damage can lead to tremors, incoordination, and inaccuracy in movements, especially in swallowing (7, 11).

Stroke is a widespread neurological deficit that affects 25% of people within their lifespan and is, respectively, the second and third significant cause of adults’ mortality and disability worldwide (12). A cerebellar infarct is a kind of stroke in which the posterior cranial fossa, in particular the cerebellum, gets involved. Cerebellar stroke has been reported to account for up to 4 % of all brain strokes (13, 14), and causes dysphagia in 11.45% of the affected population (15). Nevertheless, the effects of this infarct on the swallowing mechanism are still uncrystallized.

Although the accumulation of animal studies has revealed a prominent connection between swallowing and the cerebellum, for instance (16–21), the association between the cerebellum and swallowing has not been well explored among humans. Hence, several interventional studies have also been conducted within the last two decades to illuminate the cerebellum’s contribution to human swallowing. Accordingly, experimental studies have demonstrated that cerebellar transcranial magnetic stimulation can significantly evoke distinct pharyngeal neuromuscular responses as well as corticobulbar excitability [e.g., (22–24)]. However, two recent studies reported the opposite view. An investigation of cerebellar transcranial direct current stimulation illustrated that it can adversely affect motor skill learning in the swallowing process by causing a deterioration in the timing of submental muscle activation during swallowing (25). Furthermore, another experiment also showed that cerebellar transcranial magnetic stimulation can suppress pharyngeal motor cortical activities and swallowing behavior (26). For more information on this topic, please refer to “The Role of the Cerebellum in Swallowing” by Sasegbon and Hamdy, published in 2021 (27). They provided a comprehensive review regarding this topic. In addition to these interventional studies, several observational investigations have been accomplished and demonstrated cerebellar bilateral activation during swallowing, for example (28–30). Further, a new exploration exhibited that the posterior lobe of the left cerebellum seems to be related to dysphagia severity among persons with isolated cerebellar lesions (31). Huang et al., through a retrospective cohort study in 2023, indicated that dysphagia affected almost 11% of people with cerebellar infracts. They found higher odds ratios of dysphagia among people with mixed strokes (a combination of ischemic and hemorrhagic strokes) and with older ages. They also discovered that the higher recovery rate of swallowing disorders was related to male gender, younger adults, hemorrhagic stroke, and right cerebellum (15).

These studies presented evidence that supports the idea that the cerebellum is likely to get involved in the swallowing mechanism as also previously discussed (32). Indeed, the cerebellum may even play a crucial role in swallowing (27). Nevertheless, the exact and elaborate contribution of the cerebellum and its activities to swallowing have been poorly addressed and understood in humans, analogous to the basal ganglia (33). In fact, it seems that these studies investigated swallowing as a simple three-phase mechanism including some straightforward subsets, while just the two first stages consist of almost 20 various sub-elements that work together to provide a flawless mechanism of swallowing (34). Therefore, this study aims to determine elaborate clinical characteristics of the oral and pharyngeal phases of swallowing after isolated cerebellar stroke and reflectively describe the cerebellum’s roles and involvements within swallowing, and address how these strokes may affect the swallowing process.

## 2 Materials and methods

All gathered participants received informed consent, including thorough explanations about the study and the use of their data for the exploration. They were also informed that they are free to leave the research whenever they do not tend to continue taking part.

### 2.1 Participants

The present study examined 34 participants (mean age = 51.76; SD = 17.06; range = 27–82), including 13 females (mean age = 52.31; SD = 18.35; range = 29–82) and 21 males (mean age = 51.43; SD = 16.66; range = 27–81), with cerebellar stroke. All invited participants received comprehensive information about the present study through informed consent to make their decision about their voluntary participation in this research. They were also gathered through convenience sampling over 6 months in the following manner.

A team of neurologists made diagnoses based on Computed Tomography Scan (CTS) and Magnetic Resonance Imaging (MRI) to determine only ones with isolated cerebellar strokes, and not any other neurological regions or conditions at that moment or previously. Then a speech and language pathologist (SLP), with 3 years of experience working on swallowing disorders, examined them and explored their records for no history of oropharyngolaryngeal pathologies, tracheostomy, dysphagia, perception problems, or any chronic medical conditions. Individuals were excluded from the study if they were identified with each of the records mentioned.

### 2.2 Swallowing assessment

The SLP applied the Mann Assessment of Swallowing Ability (MASA) manual to assess their swallowing function.

The MASA is an efficient, accurate, and reliable swallowing assessment tool, which consists of 24 items allowing professionals to assess swallowing characteristics by which dysphagia can be determined and scored (34). The tool is also capable of categorizing dysphagia into four classes of severity (no abnormality detected ≥178 , mild 168–177, moderate 139–167, and severe ≤138 ) (34). Furthermore, the manual can provide a cut-off score for the risk of aspiration (no abnormality detected ≥170 , mild 149–169, moderate 141–148, and severe ≤140 ) (34).

The MASA can be performed over time to check the swallowing function. The sensitivity and specificity of the MASA in diagnosing dysphagia among people with stroke have been reported as 71 and 72%, respectively (34). Moreover, the MASA’s sensitivity and specificity to predict aspiration have been valued as 93 and 55%, respectively (34).

### 2.3 Statistical analysis

In the present study, the Statistical Package for the Social Sciences (SPSS®) was used to analyze the data. The Kolmogorov–Smirnov test was used to calculate the normality of age and the MASA–score distribution to apply appropriate statistical methods.

A t-test was calculated to examine differences across parametric data. Spearman’s rank correlation coefficient was calculated to examine the correlation (ρ) between non-parametric data. Moreover, the Mann–Whitney U test was applied to analyze differences across non-parametric data. Furthermore, this test was used to examine any probable differences among different categories.

The significance level was considered as ≤0.05.

## 3 Results

According to the Kolmogorov–Smirnov test, the age distribution was normal (*p* = 0.59) among the participants, but the MASA-score distribution was not normal (*p* < 0.00).

**Figure 1:**
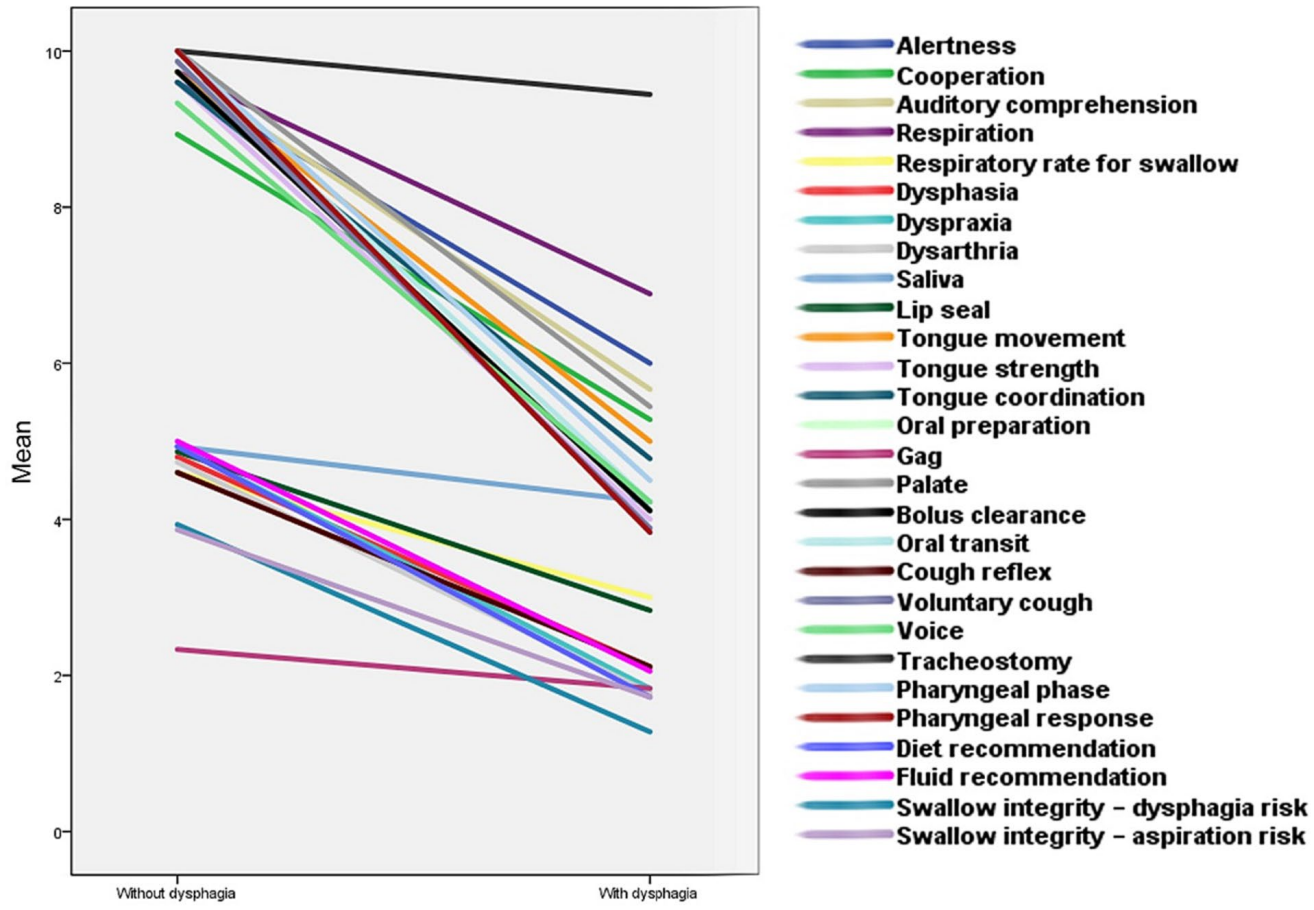
The comparison of swallowing aspects between people with and without dysphagia after cerebellar stroke. This diagram depicts swallowing characteristics between the two groups of subjects following cerebellar stroke. Almost every component of swallowing became considerably compromised among individuals with dysphagia. The slope of the lines indicates how much these factors were influenced.

Out of 34 examined participants, 16 individuals (proportion = 47.1%; mean age = 44.94; SD = 15.36; range = 29–72), including 6 females and 10 males, were identified without dysphagia, and 18 participants (proportion = 52.9%; mean age = 57.83; SD = 16.55; range = 27–82), including 7 females and 11 males, were diagnosed to have dysphagia according to the MASA scores. According to the Mann Whitney U test, swallowing ability was significantly different (*p* < 0.00) between these two groups. This difference was also significant across all MASA categories, except the gag reflex and tracheostomy categories (Table 1). Figure 1 portrays these categories and compares the differences. Indeed, the gag reflex diminished in 91.2% of all participants. Also, 94.1% of all participants had no tracheostomy tube at the time of the assessment.

**Table 1:** Comparison of MASA categories in individuals with and without dysphagia.

Category	Group	Mean	Mean rank	*U*	*p* value
Alertness	Without dysphagia	9.62	24.06	39.000	<0.00*
	With dysphagia	6.00	11.67		
Cooperation	Without dysphagia	9.00	23.12	54.000	<0.00*
	With dysphagia	5.28	12.50		
Auditory comprehension	Without dysphagia	9.75	24.00	40.000	<0.00*
	With dysphagia	5.67	11.72		
Respiration	Without dysphagia	9.75	24.25	36.000	<0.00*
	With dysphagia	6.89	11.50		
Respiratory rate (for swallow)	Without dysphagia	4.62	21.97	72.500	<0.00*
	With dysphagia	3.00	13.53		
Dysphasia	Without dysphagia	4.81	24.62	30.000	<0.00*
	With dysphagia	2.11	11.17		
Dyspraxia	Without dysphagia	4.88	24.75	28.000	<0.00*
	With dysphagia	1.83	11.06		
Dysarthria	Without dysphagia	4.75	25.62	14.000	<0.00*
	With dysphagia	1.78	10.28		
Saliva	Without dysphagia	4.94	20.59	94.500	0.02*
	With dysphagia	4.22	14.75		
Lip seal	Without dysphagia	4.88	23.94	41.000	<0.00*
	With dysphagia	2.83	11.78		
Tongue movement	Without dysphagia	9.75	25.25	20.000	<0.00*
	With dysphagia	5.00	10.61		
Tongue strength	Without dysphagia	9.62	25.94	9.000	<0.00*
	With dysphagia	4.00	10.00		
Tongue coordination	Without dysphagia	9.50	25.25	20.000	<0.00*
	With dysphagia	4.78	10.61		
Oral preparation	Without dysphagia	9.75	25.25	20.000	<0.00*
	With dysphagia	4.11	10.61		
Gag	Without dysphagia	2.25	18.31	131.000	0.59
	With dysphagia	1.83	16.78		
Palate	Without dysphagia	9.88	24.25	36.000	<0.00*
	With dysphagia	5.44	11.50		
Bolus clearance	Without dysphagia	9.75	25.75	12.000	<0.00*
	With dysphagia	4.11	10.17		
Oral transit	Without dysphagia	9.88	26.38	2.000	<0.00*
	With dysphagia	4.22	9.61		
Cough reflex	Without dysphagia	4.62	25.16	21.500	<0.00*
	With dysphagia	2.11	10.69		
Voluntary cough	Without dysphagia	9.88	25.38	18.000	<0.00*
	With dysphagia	3.89	10.50		
Voice	Without dysphagia	9.38	24.81	27.000	<0.00*
	With dysphagia	4.22	11.00		
Tracheostomy	Without dysphagia	10.00	18.50	128.000	0.17*
	With dysphagia	9.44	16.61		
Pharyngeal phase	Without dysphagia	10.00	25.00	24.000	<0.00*
	With dysphagia	4.50	10.83		
Pharyngeal response	Without dysphagia	10.00	25.00	24.000	<0.00*
	With dysphagia	3.83	10.83		
Diet recommendation	Without dysphagia	4.94	25.91	9.500	<0.00*
	With dysphagia	1.72	10.03		
Fluid recommendation	Without dysphagia	5.00	24.50	22.500	<0.00*
	With dysphagia	2.06	10.75		
Swallow integrity – dysphagia risk	Without dysphagia	3.94	26.44	1.000	<0.00*
	With dysphagia	1.28	9.56		
Swallow integrity – aspiration risk	Without dysphagia	3.88	25.69	13.000	<0.00*
	With dysphagia	1.72	10.22		

**Figure 2:**
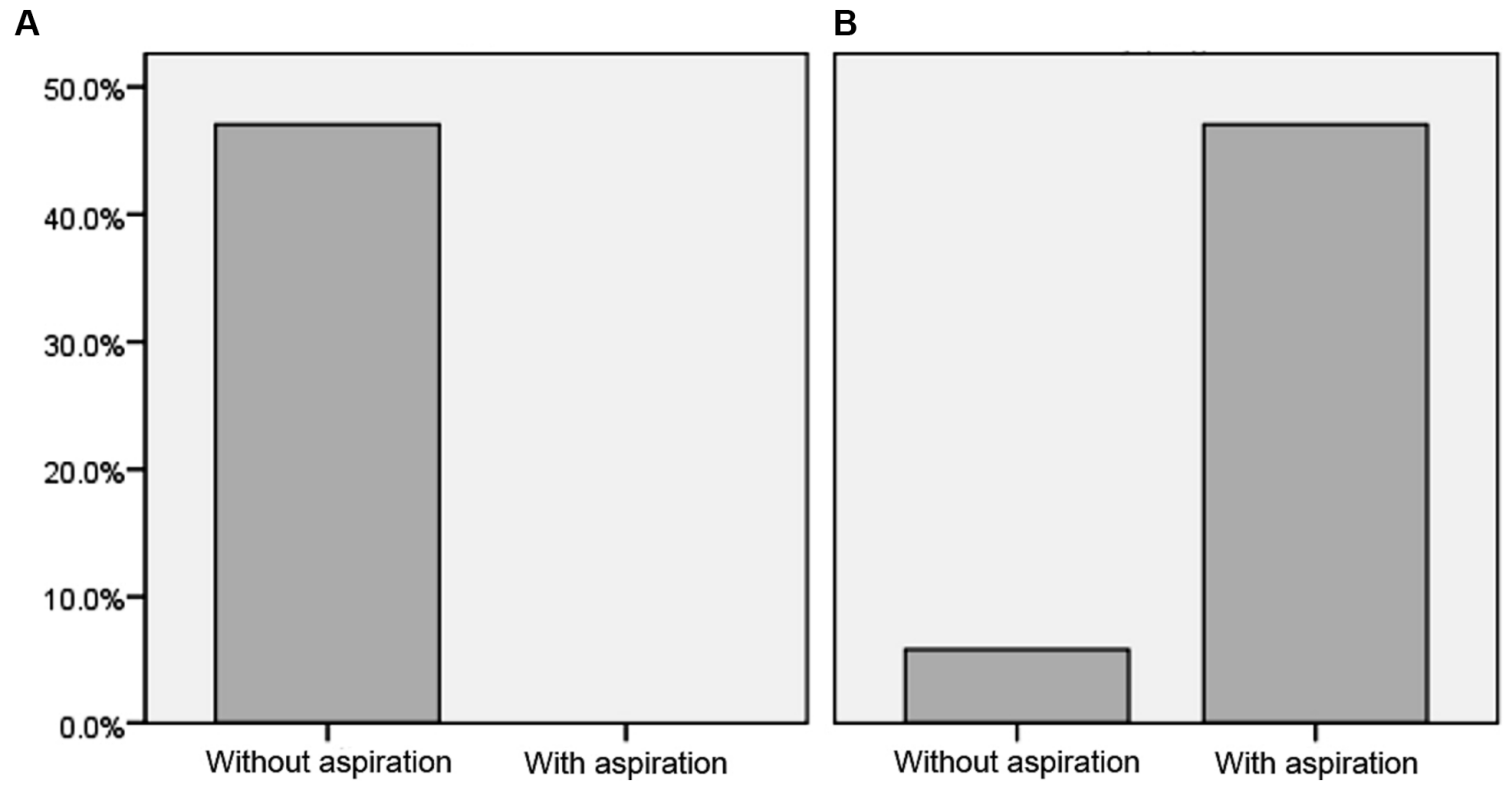
The risk of aspiration following cerebellar stroke among people with and without dysphagia. This figure illustrates the aspiration risk after cerebellar stroke. **(A,B)** show people without and with dysphagia, respectively.

16 individuals with dysphagia were identified to be at risk of aspiration (proportion = 47%; mean age = 57.12; SD = 16.27; range = 27–82). However, participants without dysphagia showed no risk of aspiration (Figure 2). Further, a significant correlation was also found between aspiration and the presence of dysphagia (*ρ* = 0.88; *p* < 0.00).

Age and the presence of dysphagia were also significantly correlated (ρ = 0.39; *p* = 0.02), and individuals with dysphagia were significantly older than people without dysphagia (df = 32; p = 0.02; CI = 95%).

No significant correlation was found between the MASA-score and sex (*p* = 0.60). Furthermore, no significant difference (*p* = 0.95) was discovered regarding the presence of dysphagia between males and females.

Table 2; Figure 2 demonstrate further details about swallowing skills and the risk of aspiration among the two groups.

**Table 2:** Swallowing ability and aspiration risk in individuals with and without dysphagia after cerebellar stroke.

	Category	Without dysphagia (%)	With dysphagia (%)	P value
Swallowing ability	no abnormality detected	16 (47.1%)	0 (0.0%)	<0.00
	mild	0 (0.0%)	2 (5.9%)	
	moderate	0 (0.0%)	3 (8.8%)	
	severe	0 (0.0%)	13 (38.25%)	
Aspiration risk	no abnormality detected	16 (47.1%)	2 (5.9%)	<0.00
	mild	0 (0.0%)	2 (5.9%)	
	moderate	0 (0.0%)	1 (2.9%)	
	severe	0 (0.0%)	13 (38.2%)	

This table clearly illustrates that swallowing disorders and the risk of aspiration were mostly severe among individuals with dysphagia following cerebellar stroke, while no abnormality was detected among those without dysphagia.

## 4 Discussion

An in-depth analysis of the clinical characteristics of the oral and pharyngeal phases of swallowing was conducted in patients with cerebellar strokes to understand how cerebellar strokes impact swallowing. The findings showed that cerebellar strokes can significantly affect the oral and pharyngeal phases of swallowing, leading to various issues such as dysphagia and aspiration.

This study indicated that almost half of the individuals who experienced a cerebellar stroke encountered dysphagia afterward. However, this is inconsistent with the results of the previous retrospective cohort study conducted in 2023, which demonstrated that dysphagia affected around 11% of individuals after cerebellar strokes (15). In the present study, people with dysphagia often underwent severe dysphagia, including prominent impairments in both oral and pharyngeal phases. Contrarily, Huang et al. could not provide detailed reports on the severity of dysphagia and the most affected phases of swallowing because they run their study through a retrospective chart review of the medical records and information reported after a swallowing bedside examination, videofuoroscopic swallowing study, fberoptic endoscopic evaluation of swallowing, etc. This might have affected the accuracy of dysphagia diagnosis after cerebellar strokes through their study. The present study suggests that the cerebellum may control both the motor and non-motor components of the swallowing process, such as tongue movements, alertness, etc. This supports the limited evidence revealing cerebellar activities during volitional swallowing among people with no medical conditions (28–30). Interestingly, an animal study has shown that the cerebellum modulates the excitability of the swallowing reflex via direct connections to the nucleus tractus solitarius (35), which has been known for its fundamental role in the oral and pharyngeal phases of swallowing (36). Although several studies have previously indicated a potential correlation between dysphagia and diseases related to the cerebellum, e.g., (37–40), ones that investigated the risk of dysphagia after isolated cerebellar strokes have reported inconsistent results with these findings (41, 42). The reason behind this can be easily understood by looking at the way these studies evaluated the process of swallowing. Indeed, the two first phases of swallowing contain various parts that need to be considered together to assess swallowing (34), but it is unclear how exactly these studies assessed swallowing since they did not provide any specific information about their procedure. Some investigations, however, utilized specific assessment methods, but they did not include an adequate number of individuals with isolated cerebellar strokes (43–45). Thus, it is not surprising that there is still uncertainty about how cerebellar strokes lead to swallowing disorders, given the limited conclusive evidence in this regard.

According to the study, individuals with dysphagia were also significantly older than those who did not have the condition. This is also in line with previous studies, which have revealed that dysphagia is common among older adults, impacting around 10 to 33% of them (46). Furthermore, Huang et al. indicated that older individuals were more likely to encounter dysphagia after cerebellar strokes (15). Evidence has demonstrated that aging can change the swallowing mechanism (47). For example, aging can affect the oral phase of swallowing by causing a reduced oral sensation, poor dentition, hyposalivation, and xerostomia, which may also make it harder to sense how thick or thin the food or liquid is in the mouth (47). Aging also causes less strength in both the masticatory muscles and the lingual muscles (48). As well as the oral phase, the laryngopharyngeal response, musculature, pressure, timing, and coordination may become weakened as people get older (49, 50). Aging can also lead to various changes in the cerebellum, which affect its size, structure, and function (51, 52). These, for example, cause a decrease in size, particularly in the anterior and posterior cerebellum, and changes in neuron density, synapse connectivity, and white matter (51). Remarkably, aging decreases the volume of the nucleus tractus solitarius too (53), which has been found to be the key that the cerebellum uses to regulate the excitability of the swallowing reflex (35). Taking it all together, the findings suggest that aging can be a key factor that determines whether people with cerebellar strokes encounter dysphagia.

As may be expected from the previous discussion, the risk of aspiration (mostly severe) was accordingly significantly higher in individuals with dysphagia, while those without dysphagia showed no risk of aspiration, which supports the close association between dysphagia and aspiration pneumonia (54). Huang et al. could not also provide precise reports about aspiration risk and its severity, according to their study design. This might have also influenced the reports and made judgment biases through their study (15). Contrarily, a high incidence of aspiration has also been reported among people after stroke in the present study, which raises the risk of pneumonia (55). Furthermore, an investigation has pointed out that hospitalized older people are even more vulnerable since aspiration pneumonia is more likely to cause or develop mortality in older people (56). Therefore, early diagnosis and management of dysphagia are imperative to preserve the optimal quality of life for the affected individuals and reduce the risk of mortality.

The present study also indicated that the gag reflex faded away in almost all participants, with or without dysphagia, after an isolated cerebellar stroke. This may imply that there may be a possible link, somehow, between the gag reflex and the cerebellum. The gag reflex is thought to be an evolutionary reflex that developed to prevent individuals from choking and swallowing foreign objects. The gag reflex is mediated by both the glossopharyngeal (CN IX) and vagus (CN X) nerves, which pass to the medulla oblongata, which is also adjacent to the vomiting, salivary, and cardiac centers. Near this area, the reticular formation is also located, which fills the spaces among cranial nerves, olivary supplies, and fiber tracts. The reticular formation reciprocally carries information from the spinal cord, cranial nerve nuclei, cerebellum, and cerebrum. Some parts of this formation are tightly connected to the respiratory centers, including the dorsal and ventral respiratory groups (57). Surprisingly, the dorsal group is part of the nucleus tractus solitarius, which is the main area for termination and integration of sensory afferents from various vital body systems, such as the soft palate, epiglottis, and pharynx. They reciprocate various sensory information through the facial (CN VII), glossopharyngeal (CN IX), and vagus (CN X) nerves. The ventral respiratory group also contains a place called the preBötzinger complex, which is located in the ventral respiratory group that encompasses respiratory premotor interneurons that regulate inspiratory related muscles of the tongue and pharynx (58–61). Investigations have reported that this site gets involved in the reflexes provoked by the vestibular (CN VIII) and (superior) laryngeal (CN X) nerves, and in swallowing and vomiting (62, 63). Furthermore, the reticular formation also has projections to the cerebellum, which may imply the integrative role of these structures in pharyngeal movements (64–67). Interestingly, the cells forming spinal projections to the reticular formation are also placed mainly in the laminae of the abducens (CN VI), facial (CN VII), and vagus (CN X) nerves, where many last order premotor interneurons are also organized. Therefore, it seems that the cerebellum may indirectly receive tremendous information through a huge complex path called the spino-bulbar-reticular pathway (68), in order to regulate various motor adjustments with the help of these structures (69). Accordingly, a cerebellar stroke could possibly result in disruptions to the cerebellum’s function and also its connected structures, such as the reticular formation, by which the gag reflex faded away. This might be an explanation for why the gag reflex was found to be substantially poor among participants. However, further research is required to study these connections and their underlying mechanisms more precisely.

In conclusion, the findings of the present study support the notion that the cerebellum presumably plays a functional role in regulating the motor and non-motor aspects of swallowing. Accordingly, isolated cerebellar strokes can adversely affect both oral and pharyngeal phases of the swallowing process, particularly among older adults. Thus, dysphagia after a cerebellar stroke should be diagnosed early and timely managed, as it causes serious consequences resulting from aspiration.

## 5 Limitations and direction for the future study

Despite the present study offering promising evidence regarding dysphagia following cerebellar strokes, there are some limitations that should be factored in. First, we did not have access to the Videofluoroscopic Swallow Study in order to assess swallowing. This is currently the gold standard for diagnosing dysphagia (70). Second, this study was run through a master’s thesis; hence, effort was made to recruit as many participants as feasible within the time and resource constraints of the project. While a larger sample would be ideal, the researchers believe these findings still provide valuable preliminary insights that can inform future research. Therefore, the results of this study should be interpreted with caution. Third, we did not consider different conditions of stroke (ischemic, hemorrhagic, or mixed) in the present study. Thus, similar studies should be carried out taking these points into consideration in different regions of the world to study the effects of cerebellar strokes on the swallowing process in different populations. Although various medical conditions like swallowing disorders have been studied after cerebral strokes (e.g., (71, 72)), the role of the cerebellum in the swallowing process and the effects of its infracts on this process still need more illumination. Hence, further studies appear to be necessary for the expansion of sufficient practical information (73).

## Data availability statement

The raw data supporting the conclusions of this article will be made available by the authors, without undue reservation.

## Ethics statement

The studies involving humans were approved by the Ethics Committee of Mashhad University of Medical Sciences with the ethical code of IR.MUMS.REC.1398.286. The studies were conducted in accordance with the local legislation and institutional requirements. Written informed consent for participation in this study was provided by the participants’ legal guardians/next of kin. Written informed consent was obtained from the individual(s) for the publication of any potentially identifiable images or data included in this article.

## Author contributions

MH: Conceptualization, Writing – original draft, Data curation. SZ: Writing – original draft, Writing – review & editing, Methodology, Formal Analysis, Visualization. MF: Investigation, Writing – review & editing. SK: Formal analysis, Writing – review & editing. DS-R: Conceptualization, Supervision, Writing – original draft, Writing – review & editing.
